# Seed Germination and Urban Wastewater Remediation Using Micro-Fe-Modified Montmorillonite

**DOI:** 10.1021/acsomega.5c04144

**Published:** 2025-09-30

**Authors:** Wagner A. Carvalho, Josefina Schmuck, Gabriela T. M. Xavier, Natalí Romero, Pedro S. Fadini, Ana M. Gagneten

**Affiliations:** 1 Center for Natural and Human Sciences (CCNH), 74362 Universidade Federal do ABC, Santo André, Avenida dos Estados 5001, São Paulo 09280-560, Brazil; 2 Laboratorio de Ecotoxicología, Facultad de Humanidades y Ciencias, 28247 Universidad Nacional del Litoral, RN 168, Km 0, Santa Fe S3000ADQ, Argentina; 3 CONICET, Santa Fe, RN 168, Km 0, Santa Fe S3000ADQ, Argentina; 4 Laboratorio de Biogeoquìmica Ambiental, Universidad Federal de São Carlos, Rodovia Washington Luís, km 235, São Carlos, São Paulo 13565-905, Brazil

## Abstract

In recent years, it has become increasingly clear that agricultural production must adopt innovative and sustainable strategies to enhance productivity, while reducing environmental harm. Urban wastewater, rich in phosphorus (P), represents a valuable yet underused resource for agriculture, offering an alternative to the continuous addition of synthetic fertilizer input. This research explores a nanotechnology-based solution utilizing a montmorillonite composite enriched with Fe (TechPhos) to enhance germination. XRD, XRF, and N_2_ adsorption/desorption isotherms characterized TechPhos. Then, the effects of TechPhos on the germination process of lettuce (*Lactuca sativa*), soy (*Glycine max*), and rice (*Oryza sativa*) after exposure to 0.4 g L^–1^ TechPhos were assessed. Seeds in the TechPhos treatment germinated rapidly, reaching 82–100, 73–100, and 57–95% germination for *L. sativa*, *G. max*, and *O. sativa*, respectively. The germination index remained relatively stable in lettuce, while in soy, it peaked at 48 h before gradually decreasing, and in rice, it showed a declining trend. Germination velocity and mean germination time (MGT) followed comparable trends. For *L. sativa*, the MGT showed a gradual increase in the control and with TechPhos, reaching 5.0 ± 0.0 at 120 h in the control and 4.9 ± 0.1 under the TechPhos treatment. In *G. max*, the MGT exhibited a similar trend in both conditions, increasing from 0.7 ± 0.1 at 24 h to 5.0 ± 0.0 at 120 h. For *O. sativa*, the MGT was 1.1 ± 0.3 at 48 and 4.6 ± 0.1 at 120 h in the control compared to 1.4 ± 0.1 at 48 h and 4.8 ± 0.1 at 120 h under the TechPhos treatment. *L. sativa* showed a 35% increase in root elongation (*p* < 0.001) and a 37% increase in hypocotyl elongation (*p* < 0.001) under TechPhos treatment. At the same time, *G. max* exhibited a smaller but significant increase in root length (*p* < 0.05). Our findings indicate that TechPhos is not toxic to the three species, enhances germination and root growth of *L. sativa* and *G. max* seeds, and allows its use as fertilizer, offering a dual benefit: reducing nutrient pollution and strengthening their potential for agricultural applications.

## Graphical abstract

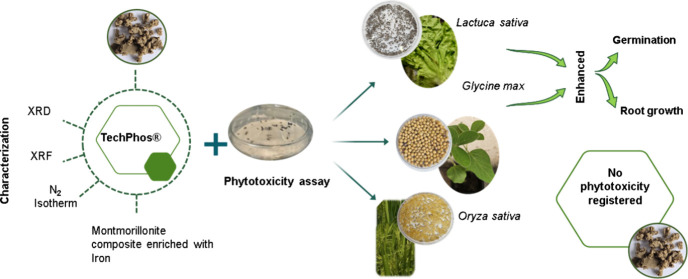

## Highlights

- Structural and chemical characterization of TechPhos
- TechPhos successfully applied in tertiary wastewater treatment and phosphorus recovery
- No phytotoxicity to lettuce, soy, and rice
- Enhanced germination and root growth of *L. sativa* and *G. max* seeds
- TechPhos-sludge composite, as a novel slow-release nutrient delivery

## 1 Introduction

The Latin America and Caribbean (LAC) region has achieved notable progress in boosting agricultural production and competitiveness.^1^ However, nutrient release remains a complex issue shaped by agricultural practices, environmental conditions, and dietary patterns. To assess the balance of key macronutrients—nitrogen (N), phosphorus (P), and potassium (K)—used by crops, Guareschi et al.^2^ analyzed nutrient consumption and export in LAC’s agricultural systems. Their findings reveal considerable variability in nutrient export, especially for N and P, which are essential not only for crop productivity but also for ecosystem sustainability.^3^ In this context, nutrient balance assessments highlight how efficiently crops utilize fertilizers as well as the extent of nutrient extraction from soils, which directly affects soil fertility. A negative nutrient balance can jeopardize long-term food security in the region.^2^ Furthermore, the rising global population has intensified pressure on agricultural systems, often resulting in the excessive application of nutrients, synthetic fertilizers, and pesticides in recent decades.^4^

Despite widespread fertilizer use, efficiency remains considerably low—over half of the N applied in the field is lost to the environment, while only 15–25% of P from fertilizers is taken up by plants.^5^ This inefficiency leads to substantial nutrient losses, ultimately reducing agricultural productivity.^6^ Moreover, it contributes to serious environmental problems, including nutrient runoff into aquatic ecosystems, which accelerates eutrophication and leads to biodiversity loss.

In light of these challenges, achieving food security with minimal environmental impact calls for innovative, sustainable agricultural practices that improve productivity while preserving ecosystems. Nanotechnology presents a promising solution, offering the potential to design nanomaterials that enable the controlled release of nutrients.^6,7^ Among the key innovations needed to meet the growing global demand for food are advanced fertilizers.^8^ Nanofertilizers, in particular, can enhance the efficiency of traditional fertilizers by 20–50%, improve nutrient solubility, and boost soil fertility.^9,10^

Among the various delivery systems explored in agriculture, nanoclays have garnered attention due to their high surface area, cation exchange capacity, and ability to protect active ingredients from rapid degradation. Nanoclay materials are cheap, nontoxic, and abundantly available in nature.^3,11^ Montmorrilonite (MnT) stands out for its superior interlaminar spacing and cation exchange capacity compared to other clays such as kaolinite.^12^ These properties make MnT an excellent vehicle for the controlled delivery of nutrients, plant growth regulators, and pesticides. TechPhos, a phosphorus-selective adsorbent composed of iron-modified natural clay, has been developed to retain phosphorus in wastewater treatment processes, offering a dual benefit: reducing nutrient pollution in urban wastewater with potential environmental issues and recycling essentials nutrients like P, N, K, Mg, Ca, Cl, and Fe, for agricultural use.^13^ Previous research suggested that in a Brazilian wastewater treatment plant, the adsorption capacity of TechPhos was 13.1 mg P g^–1^, leading to a remarkable 97.0% phosphorus removal efficiency.^13^ Beyond removing nutrients from domestic effluents to protect the environment, their reuse is equally—if not more—important. For this purpose, it is essential to maintain their bioavailability for plants. However, adsorbent safety and efficacy must be evaluated before considering its use as a nanomaterial in agriculture. Germination assays are widely recognized as an effective method to assess the potential impacts of new materials on plant development. In particular, the seed germination and root elongation tests are one of the simplest methods for phytotoxicity testing,^14–16^ because the germination seed is the first interface of material exchange between the developing plant and the environment.^17^

Lettuce (*Lactuca sativa*), soy (*Glycine max*), and rice (*Oryza sativa*) were selected as model species for this study due to their economic relevance and sensitivity to environmental conditions. *L. sativa* is highly sensitive to contaminants and has been endorsed by international organizations,^18–20^ and other studies, for assessing the ecological effects of toxic substances and conducting standard toxicity tests.^21,22^ This makes it an ideal candidate for evaluating the impact of innovative materials, such as TechPhos, on plant development.

*G. max* is valued for its high protein and oil content and is a crucial crop, with the United States, Brazil, and Argentina leading the global market.^23,24^ Brazil is the second largest soybean producer in the world with a planted area in the crop year 2017/2018 of 33.347 million hectares,^25^ while the National Supply Company (Conab) estimates that, in the 2024/2025 harvest, soybean production will reach 167.4 million tons. *O. sativa* is one of the most important staple crops globally, playing a crucial role in food security, nutrition, and the economy. Providing a primary source of calories for more than half of the world′s population, Brazil is the largest producer and consumer of rice outside Asia.^26,27^

Seed germination tests have been intensively used globally; however, no comprehensive studies have been conducted to assess the effects of montmorillonite modified with Fe on staple food species. We suggest that the novel Fe-enriched montmorillonite composite (TechPhos) may improve the germination and growth of lettuce, soy, and rice seeds.

TechPhos is a modified clay-based adsorbent for removing P from wastewater treatment plants (WWTP), whose technology was developed by a Brazilian research group from the Federal University of São Carlos and the Federal University of ABC (patent BR 102020018920-4). The goal was to utilize inexpensive and abundant raw materials to create a solid with high and selective ion exchange capacity, strong resistance to environmental conditions, and ability to release the adsorbed nutrient in a controlled manner. The adsorbent has been tested in the treatment of effluents from wastewater treatment plants, both on a bench scale and in field tests, presenting very satisfactory results such as those that will be presented in this work.

This study aims to (i) characterize TechPhos using X-ray diffraction (XRD), X-ray fluorescence (XRF), and N_2_ adsorption/desorption isotherms and (ii) assess its potential as a nanofertilizer by evaluating its effects on the germination and growth of *L. sativa*, *G. max*, and *O. sativa*. In addition, its large-scale application is proposed in combination with sewage treatment plant sludge to assess the effects of seed treatment not only on germination but also on the entire plant growth and development process. This approach introduces an innovative slow-release nutrient delivery system aligned with the principles of sustainable agriculture.

## 2 Materials and Methods

### 2.1 TechPhos General Characterization

The TechPhos was prepared following the protocol described in patent BR 102020018920-4 and characterized using X-ray diffraction (XRD), X-ray fluorescence (XRF), and N_2_ adsorption/desorption isotherms to evaluate its physicochemical properties.

XRD analysis was performed using a Bruker D8 FOCUS diffractometer (São Paulo, Brazil). Diffractograms were collected over a 2θ range from 5 to 90° with a step size of 0.015°. XRF analyses of montmorillonite clay (MnT) and iron-modified clay (TechPhos) were performed using a Supermini200 high-power benchtop sequential wavelength dispersive X-ray fluorescence spectrometer from Rigaku, at 50 kV and 4 mA. The samples were finely ground and pressed before the analyses. N_2_ adsorption/desorption isotherms were obtained in an Autosorb-1-MP device (Quantachrome Instruments) at 77 K. Before each measurement, 100 mg of each sample was degassed at 150 °C for 4 h. The specific surface area was estimated by the Brunauer–Emmett–Teller (BET) method. The total pore volume was obtained at a relative pressure of P/P0 = 0.95, and the micropore volume was determined by using the Dubinin–Radushkevich (DR) method.

### 2.2 Phosphorus Removal from Aqueous Effluent

Phosphorus removal was assessed using a Jartest procedure applied to an effluent from a prolonged aeration-activated sludge treatment system. The chemical characteristics of the effluent before and after WWTP (treatment with and without the addition of TechPhos) (Table 1).

**Table 1:** Chemical Characteristics of the Effluent before and after WWTP (Treatment with and without the Addition of TechPhos)

component	original effluent	treated effluent without TechPhos	treated effluent with TechPhos
total biochemical oxygen demand (BOD)	428 mg/L	7 mg/L	4 mg/L
total chemical oxygen demand (COD)	803 mg/L	37 mg/L	37 mg/L
total phosphorus	4.6 mg/L	1.1 mg/L	0.4 mg/L
total suspended solids		<30 mg/L	<30 mg/L
total aluminum		1.3 mg/L	2.7 mg/L
turbidity	48.5 NTU	2.7 NTU	1.7 NTU
Kjeldahl nitrogen	49.6 mg/L	3.8 mg/L	4.7 mg/L
nitrogen as ammonia	37.6 mg/L	2.9 mg/L	2.3 mg/L
nitrogen as nitrate	41.5 mg/L	19.7 mg/L	17.8 mg/L
pH	7.58	6.95	7.00

A dosage of 0.2 g of adsorbent per liter of effluent was used. The suspension was subjected to rapid mixing at 300 rpm for 10 s to achieve a uniform dispersion of the adsorbent, followed by slow mixing at 60 rpm for 30 min to facilitate adsorption processes. Subsequently, the mixture was allowed to settle under quiescent conditions for 30 min. After sedimentation, supernatant samples were collected for analysis.

The total phosphorus concentration in the supernatant was determined using the ascorbic acid method, following the Standard Methods for the Examination of Water and Wastewater, according to Method 4500-P E,^28^ with measurements performed on a Hanna photometer, model HI83399. The turbidity of the samples was also measured according to the Nephelometric method 2130B,^28^ using a Hanna turbidimeter model HI93703.

### 2.3 Toxicity Tests

Germination and root elongation toxicity tests with *L. sativa*, *G. max*, and *O. sativa* seeds were carried out following Greene et al.,^29^ and the guidelines seed analysis rules were published by the Ministério da Agricultura, Pecuária e Abastecimento – Brazil.^30^ The seeds were sourced as follows: Lettuce seeds were purchased from Isla Sementes LTDA, soy seeds were purchased from Battaglia SRL, DM #52R19, Cod.23. Coop.053.017 (Rosario, Argentina), and rice seeds were obtained from Mercado Municipal de Pinheiros (São Paulo, Brazil). The seeds were surface-sterilized by 2% v/v H_2_O_2_ for 15 min followed by thorough washing in deionized water. The tests were performed under static conditions using 100 mm glass Petri dishes for each replicate. Previous unpublished data indicated that the concentration of 0.2 g L^–1^ TechPhos was not phytotoxic to *L. sativa*, yielding a Germination Index of 96%, with no significant differences compared to the control. Based on these results, the current study tested a higher concentration (0.4 g L^–1^) and a negative control (no TechPhos) on all three seed types, in triplicate, for a period of 120 h.

The test solution consisted of reconstituted hard water as the suspension medium, composed of CaCl_2_·2H_2_O—294.0 mg, MgSO_4_·7H_2_O—123.3 mg, NaHCO_3_—64.8 mg, and KCl—5.75 mg, per liter of deionized water.^31^ The suspension was stirred with a magnetic stirrer for 15 min to ensure good dispersion. For *L. sativa* and *O. sativa* assays, each Petri dish, lined with filter paper, contained 20 randomly distributed seeds, resulting in a total of 60 seeds per species for both the control and the TechPhos suspension treatments. For the bigger *G. max* seed assays, only five seeds were randomly placed for each Petri dish, counting four plates per replicate. This assay was also conducted in triplicate, using a total of 60 soy seeds for both the control and the TechPhos suspension treatments. For uniformity, the seeds were arranged on the plates with consistent growth spacing. In each plate, 3 mL of TechPhos suspension at a concentration of 0.4 g L^–1^ and 3 mL of the reconstituted hard water for negative controls were added. Plates were covered with aluminum foil to minimize evaporation and incubated in the dark at 21 ± 1 °C and 80% humidity in an incubation chamber to ensure constant conditions. After 120 h, the germinated seeds were exposed to light for an additional 48 h to enhance hypocotyl development, as elongation is regulated by light-responsive signaling pathways.^32^ The acceptability of the results was a percentage germination >90% in the control.^28^

### 2.4 End-Point Evaluation and Data Analysis

The potential beneficial or detrimental effects of TechPhos on the germination and growth of *L. sativa*, *G. max*, and *O. sativa* seeds were assessed through the following end points: (1) radicle elongation (mm), (2) hypocotyl elongation (mm), both determined using the Fiji Software,^33^ (3) germination percentage (GP%; eq 1), (4) germination velocity (GV; eq 2), (4) mean germination time (MGT; eq 3), and (5) germination index (GI; eq 4). For image analysis, the radicle and hypocotyl microphotographs were imported into the ImageJ program.

$$\mathrm{GP}\left(\%\right)=\frac{\mathrm{number}\;\mathrm{of}\;\mathrm{seeds}\;\mathrm{germinated}}{\mathrm{number}\;\mathrm{of}\;\mathrm{total}\;\mathrm{seeds}\in \mathrm{plate}}\times 100$$ 1

$$\mathrm{GV}=\frac{1}{\mathrm{average}\;\mathrm{germination}\;\mathrm{per}\;\mathrm{day}}\times 100$$ 2

$$\mathrm{MGT}=\frac{\sum N_{i}\times T_{i}}{N}$$ 3

where *N_i_* is the number of seeds germinated for each day, *T_i_* is the number of days after sowing, and *N* is the total number of seeds germinated at the termination of the experiment.^34^

$$\mathrm{GI}=\frac{\left[\frac{X_{\mathrm{gs}}}{X_{\mathrm{gc}}}\times 100\right]\times \left[\frac{X_{\mathrm{rt}}}{X_{\mathrm{rc}}}\times 100\right]}{100}$$ 4

where *X*_gs_ is the arithmetic mean of the number of germinated seeds in the treated sample, *X*_gc_ is the arithmetic mean of the number of germinated seeds in the control, *X*_rt_ is the arithmetic mean of the root length of the treated sample, and *X*_rc_ is the arithmetic mean of the root length of the control.^35^

The phytotoxicity level of the treated samples with TechPhos was determined using the germination index and classified based on the criteria described in Shafique et al.^34^

### 2.5 Statistical Analysis

All biological data obtained from *L. sativa*, *G. max*, and *O. sativa* assays were analyzed using Welch’s *t* test after verifying normality with the Shapiro–Wilk test and homoscedasticity of variances with Levene’s test. For nonparametric data, the Mann–Whitney test was used. For statistical analysis and data visualization, JASP version 0.18.3 and OriginPro version 9 software was used.

## 3 Results

### 3.1 TechPhos Physicochemical Characterization

The XRD patterns of natural clay MnT and iron-modified clay TechPhos revealed a heterogeneous mineral composition, including cristobalite, quartz, and montmorillonite (Figure 1). In addition, it is possible to observe peaks related to the presence of iron minerals, in the form of goethite (α-FeO­(OH)) and hematite (Fe_2_O_3_), cristobalite (SiO_2_), and titanium dioxide in the form of anatase (TiO_2_).^36,37^

**Figure 1:**
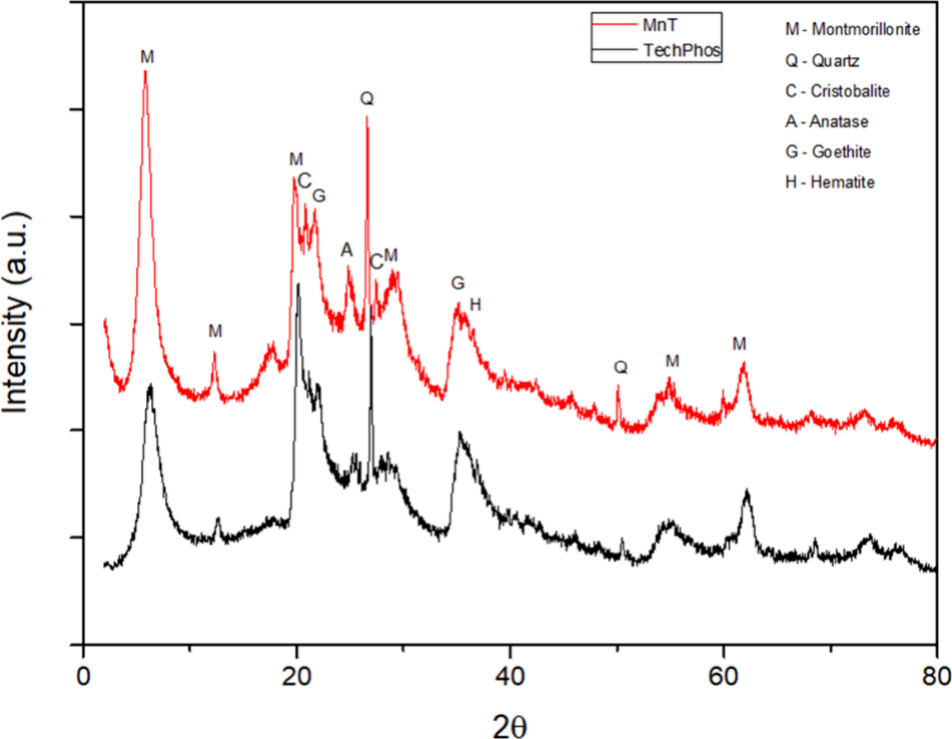
X-ray diffractograms of montmorillonite clay (MnT) and iron-modified clay (TechPhos).

The variation in the diffraction angle *d*_001_ of montmorillonite-type clays reflects the expansion or contraction of the interlayer space due to differences in the exchangeable cations. Since MnT is a natural material with a poorly crystalline structure, the diffractogram peaks were relatively broad. The interatomic distances calculated using Bragg’s equation were 15.1 Å for MnT and 14.1 Å for TechPhos, indicating that the interlayer cation significantly influences the basal spacing of the clay. For MnT, the observed spacing is characteristic of smectites with calcium as the dominant interlayer cation. The reduction in basal spacing from the original clay to the iron-modified clay is attributed to the smaller ionic radius of Fe­(III) (0.65 Å) compared to Ca­(II) (0.99 Å) and the stronger attraction of Fe­(III) ions with the silicate structure.^38,39^

XRF analysis (Table 2) confirmed that MnT and TechPhos are bentonites composed primarily of silicon and aluminum, with calcium as the dominant exchangeable cation. The results also verified the effectiveness of the ion exchange process in substituting Ca­(II) with Fe­(III). In MnT, iron can exist either as an accessory mineral (e.g., iron oxide) or within bentonite’s structural interstices of bentonite, acting as an exchangeable or structural cation.^40,41^ In the case of TechPhos, a significant reduction in the calcium content and a corresponding increase in the iron content confirm the success of the cation exchange process, where calcium has been effectively replaced by iron.^42^

**Table 2:** X-ray Fluorescence (XRF) Analysis of Montmorillonite Clay (MnT) and Iron-Modified Clay (TechPhos) Concentrations Expressed in Weight Percent (wt %)

component	MnT wt %	TechPhos wt %
SiO_2_	60.74	59.43
Al_2_O_3_	19.05	18.26
Fe_2_O_3_	12.92	17.90
MgO	2.61	1.77
CaO	1.71	0.04
TiO_2_	1.15	1.41
K_2_O	1.08	1.01
Cl^–^	0.23	
P_2_O_5_	0.19	
SO_3_	0.18	0.01
Cr_2_O_3_	0.02	
SrO	0.02	
ZnO	0.02	0.02
MnO	0.01	

The surface area and pore size of natural MnT clay and TechPhos were determined using Brunauer–Emmett–Teller (BET) and density functional theory (DFT) methods. The specific surface area was calculated as the total surface area of the solid per unit mass. Micro- and mesoporous volumes represent pores smaller than 2 nm and between 2 and 50 nm, respectively. The specific surface area was calculated using the Brunauer–Emmett–Teller (BET) method in the relative pressure range of *P*/*P*0 = 0.05–0.30, assuming multilayer adsorption on a homogeneous surface. The BET equation was applied to determine the monolayer capacity from which the surface area was obtained using the cross-sectional area of the nitrogen molecule.

The pore size distribution and total pore volume were derived from the adsorption branch of the isotherm using density functional theory (DFT) models, assuming a [insert model type, e.g., slit-shaped pore geometry. The total pore volume was estimated from the amount of nitrogen adsorbed at a relative pressure of *P*/*P*0 ≈ 0.99, and the average pore diameter was calculated based on the DFT-derived distribution. The results are summarized in Table 3.

**Table 3:** Average Values and Standard Deviation, of Specific Surface Area (*S*_BET_), Micropore Volume (*V*_micro_), Mesopore Volume (*V*_meso_), Total Pore Volume (*V*_total_) of Montmorillonite Clay (MnT), and Iron-Modified Clay (TechPhos)

sample	*S*_BET_ (m^2^/g)	*V*_micro_ (cm^3^/g)	*V*_meso_ (cm^3^/g)	*V*_total_ (cm^3^/g)
MnT	88.1 ± 4.0	0.038 ± 0.002	0.039 ± 0.002	0.077 ± 0.01
TechPhos	115.9 ± 5.1	0.050 ± 0.002	0.055 ± 0.002	0.105 ± 0.01

Statistical analysis indicates that the differences between MnT and TechPhos are significant (*p* < 0.05) for specific surface area, micropore volume, and mesopore volume. These results confirm that TechPhos exhibits higher surface area and pore volume compared to MnT.

The nitrogen adsorption–desorption isotherms (Figure 2) exhibited a type IV profile, characteristic of micro- and mesoporous materials, according to the IUPAC classification.^43^ Additionally, a type H3 hysteresis loop, typical of lamellar materials such as clays and lacking a plateau at high *P*/*P*0 values, was observed.

**Figure 2:**
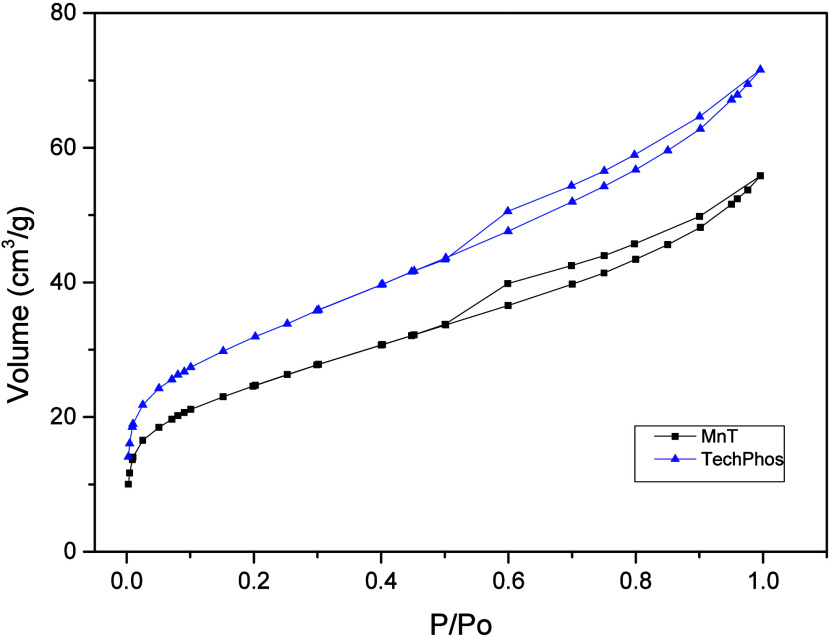
Nitrogen adsorption and desorption isotherms of montmorillonite clay (MnT) and iron-modified clay (TechPhos).

### 3.2 Phosphorus Removal from Aqueous Effluent

TechPhos was applied to remove phosphorus from the effluent of a sewage treatment plant, which initially exhibited a phosphorus concentration of 4.6 mg of P L^–1^, turbidity of 48.5 NTU, and pH 7. After a contact time of 1 h, a phosphorus removal efficiency of 90.3% was achieved, resulting in a final concentration of 0.4 mg of P L^–1^. This corresponded to a phosphorus uptake capacity (*q*) of 18.9 mg of P g^–1^, using a TechPhos dosage of 0.22 g L^–1^. By comparison, the conventional treatment process, without TechPhos, typically achieved a phosphorus removal efficiency lower than 76%.

In addition to phosphorus removal, the application of TechPhos also led to a significant reduction in turbidity, from 48.5 to 1.7 NTU. This turbidity reduction is attributed to the removal of organic matter through surface interactions with the adsorbent. The organic matter present in the effluent mainly consists of proteins, carbohydrates, lipids, and surfactants, which interact with the adsorbent surface via multiple mechanisms.^43^ TechPhos facilitates the aggregation and removal of fine organic particles that are typically difficult to eliminate through conventional treatment processes. Consequently, both the phosphorus concentration and turbidity were significantly reduced in the treated effluent.

### 3.3 Toxicity Test

#### 3.3.1 Germination

The administration of TechPhos suspension (0.4 g L^–1^) and the APHA medium (negative control) to *L. sativa*, *G. max*, and *O. sativa* seeds induced rapid germination from the second day of the experiment.

For *L. sativa*, in the control group, the GP% reached 87% at 24 h and 100% from 48 h onward, while under TechPhos treatment, it progressed from 82% at 24 h to 98% at 120 h. The GI remained relatively stable, increasing slightly from 127.3 at 24 h to 132.8 at 96 h, maintaining this value until the end of the trial (Figure 3A). The GV was 5.8 at 24 h and stabilized at 5.0 thereafter. Under TechPhos treatment, GV started higher (6.1 at 24 h) and gradually decreased to 5.1 at 96 h, remaining unchanged until 120 h (Figure 3D). The MGT showed a gradual increase in both conditions, reaching 5.0 ± 0.0 at 120 h in the control and 4.9 ± 0.1 under the TechPhos treatment (Figure 3G).

**Figure 3:**
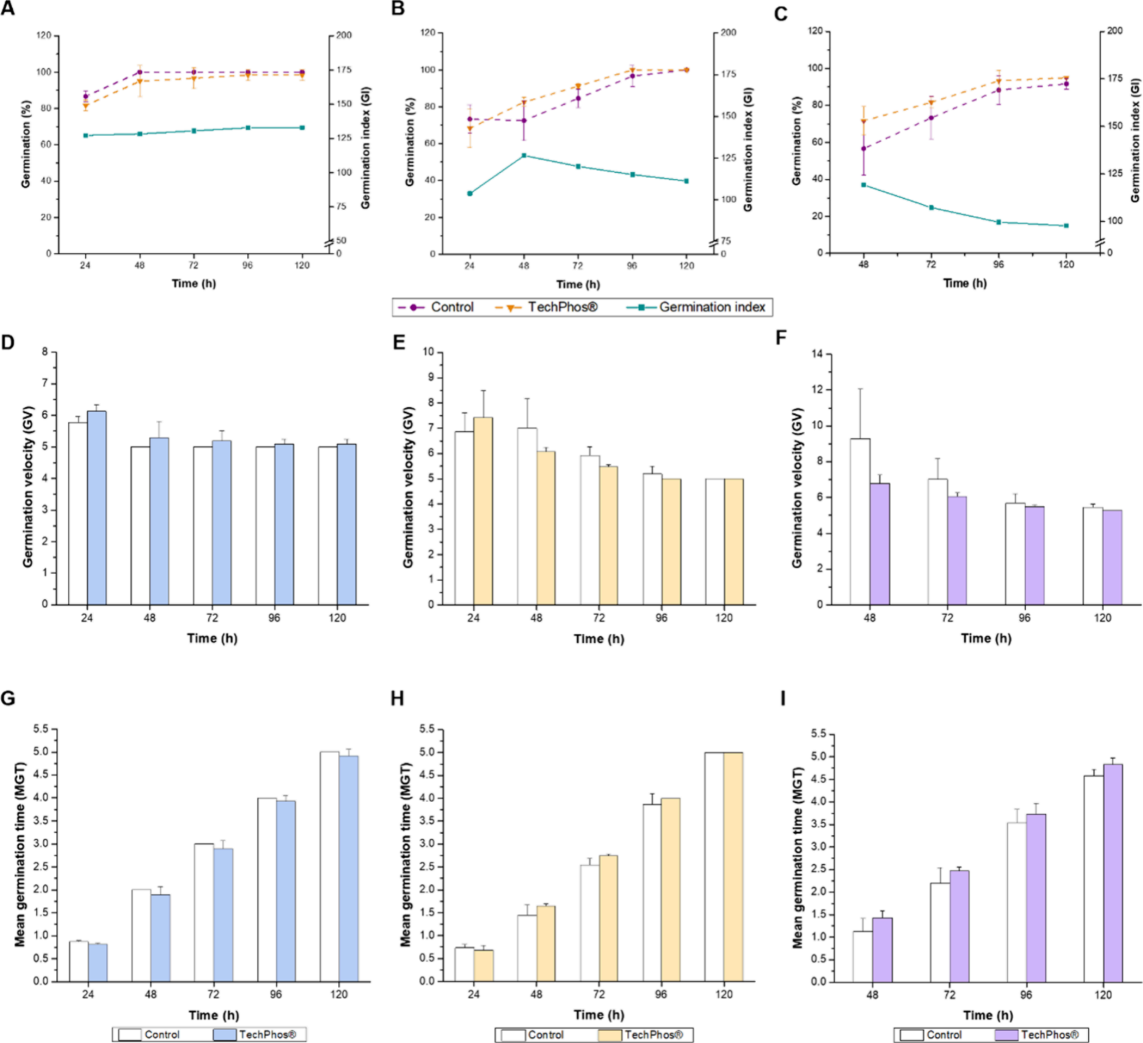
Germination percentages (GP%) and germination index (GI), germination velocity (GV), and mean germination time (MGT) of *Lactuca sativa* (A, D, G), *Glycine max* (B, E, H), and *Oryza sativa* (C, F, I) seeds exposed to APHA medium (negative control) and TechPhos (0.4 g L^–1^) at 24, 48, 72, 96, and 120 h. Data are presented as means, and error bars represent ± standard deviation (*n* = 3). Significant differences at *p* < 0.05.

In the case of *G. max*, control seeds exhibited GP% values of 73% at 24 h, reaching 97 and 100% at 96 and 120 h, respectively. Seeds under TechPhos followed a slower germination pattern, starting at 68% at 24 h and also reaching 100% at 120 h (Figure 3B). The GI showed 103.6 at 24 h and increased to 126.5 at 48 h and then decreased steadily to 119.9, 115.0, and 111.2 at 72, 96, and 120 h, respectively (Figure 3B). The GV in the control remained relatively stable, varying between 6.8 and 5.0 at 24 to 120 h. In the TechPhos treatment, it started higher at 7.3 at 24 h but progressively aligned with the control values at 5.0 at 120 h (Figure 3E). The MGT followed a similar trend in both conditions, increasing from 0.7 ± 0.1 at 24 h to 5.0 ± 0.0 at 120 h in the control and in the TechPhos treatment (Figure 3H).

For *O. sativa*, control seeds showed a GP% of 57% at 24 h, increasing to 92% at 120 h. Seeds under TechPhos exhibited a slightly delayed response, but overall positive response, reaching 72% at 48 h and 95% at 120 h. Despite this delay, the treatment still promoted germination. The GI followed a decreasing trend, starting at 91.7 at 24 h, 107.3 at 48 h, and dropping to 97.7 at 120 h (Figure 3C). The GV in the control declined from 8.8 at 48 h to 5.4 at 120 h, while under the TechPhos treatment, it was initially higher with 7.0 at 24 h and 5.3 at 120 h (Figure 3F). The MGT was 1.1 ± 0.3 at 48 h and 4.6 ± 0.1 at 120 h in the control and 1.4 ± 0.1 at 48 h and 4.8 ± 0.1 at 120 h in the TechPhos treatment (Figure 3I).

#### 3.2.2 Radicle Elongation and Hypocotyl Elongation

The average root elongation in the control group was 11.3 ± 3.2 mm of *L. sativa*, 12.8 ± 3.4 mm for *G. max*, and 15.6 ± 4.3 mm for *O. sativa* roots. Under TechPhos treatment, these values increased to 15.3 ± 4.2, 14.2 ± 4.0, and 14.7 ± 4.6 mm, respectively (Figure 4A). Statistically significant differences compared to the control were found for *L. sativa* (*p* < 0.001), with a 35% increase, and for *G. max* (*p* < 0.05), with an 11% increase.

**Figure 4:**
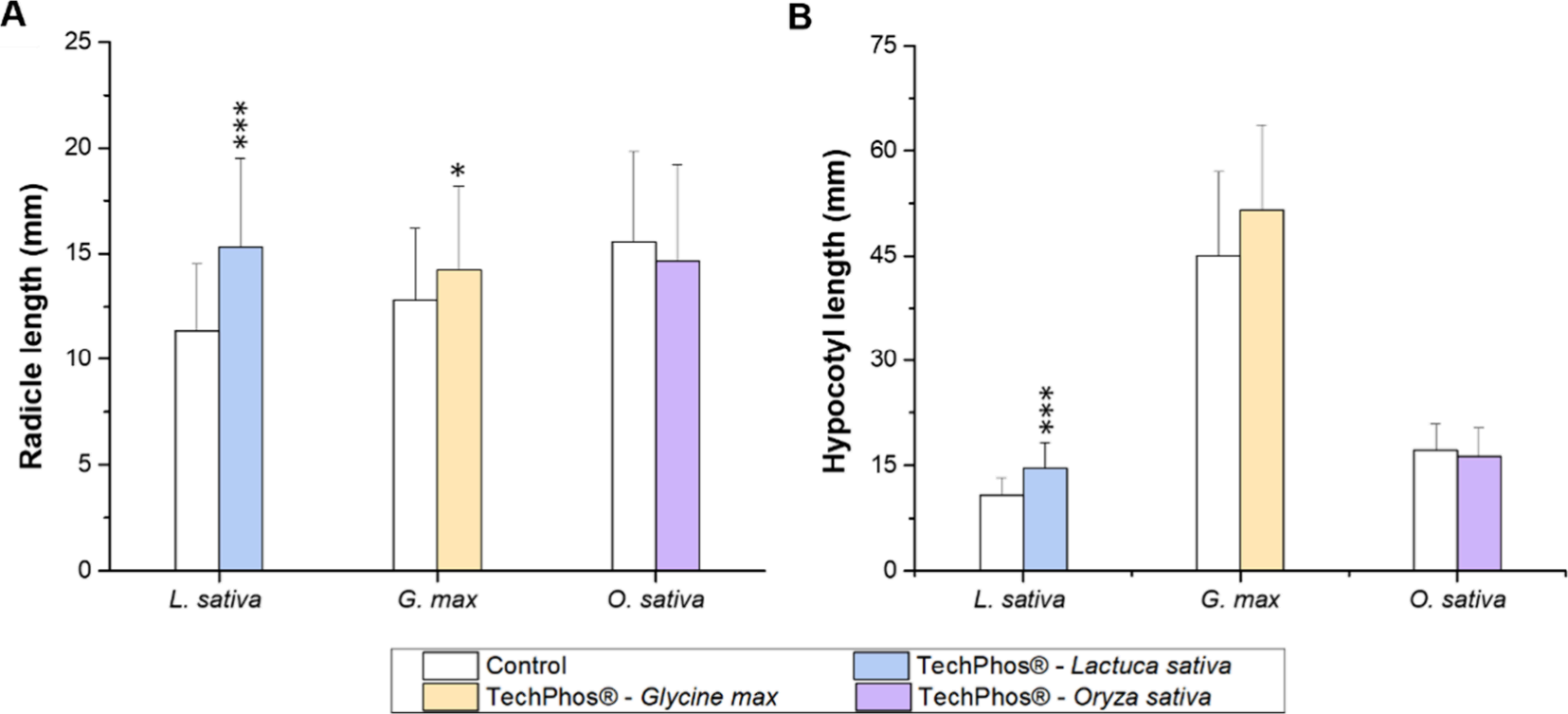
(A) Average radicle length (mm) and (B) average hypocotyl length (mm) of *Lactuca sativa*, *Glycine max*, and *Oryza sativa* germinated in APHA medium (negative control) and TechPhos (0.4 g L^–1^). Error bars represent the standard deviation (*n* = 3). Significant differences: *p < 0.05, ***p < 0.001.

The average hypocotyl elongation in the control group was 10.7 ± 2.6 mm for *L. sativa*, 45.1 ± 12.1 mm for *G. max,* and 17.1 ± 3.7 mm for *O. sativa*. In the TechPhos treatment, the corresponding values were 14.6 ± 3.5, 51.5 ± 12.2, and 16.3 ± 4.0 mm for *L. sativa*, *G. max*, and *O. sativa*, respectively. A statistically significant increase was observed in *L. sativa* compared to the control (*p* < 0.001), with a 37% elongation increment (Figure 4B).

Considering Shafique et al.'s^33^ classification (Table 4), 0.4 g L^–1^ TechPhos is not phytotoxic to *O. sativa* and it enhances germination and root growth of *L. sativa* and *G. max* seeds.

**Table 4:** Germination Index Classification^a^

%GI classification of the material under analysis	
<30	high phytotoxicity
30–60	phytotoxic
60–80	moderately phytotoxic
80–100	nonphytotoxic
>100	the tested material enhances germination and root growth of seeds

a Modified from Shafique et al.^34^

## 4 Discussion

The growing demand for food, driven by an increasing world population and decreasing availability of arable land, poses significant challenges for modern agriculture.^44^ Specifically in the LAC region, the challenge is how to bridge the gap that exists between being an agriculture powerhouse and facing persistent nutrition problems from the same households that produce the food, along with the development of new materials and waste management.^45^

Regarding phosphorus, although the LAC region has an overall positive P balance—mainly due to the large contribution of fertilized soils from Brazil—some countries, including Argentina, Bolivia, Guatemala, and Mexico, show a negative balance, leading to soil impoverishing. This situation is particularly concerning in Argentina, one of the world’s largest agricultural producers. There, large production areas rely on naturally fertile soils with low crop responses to fertilization, resulting in low fertilization rates and frequent depletion of soil nutrients.^2^

To address these challenges, many countries have invested in agricultural research and technology to improve productivity and climate resilience. These efforts include optimizing cultivation practices to sustain their role as leading producers and exporters. Various initiatives have been recognized as promising solutions for advancing agricultural technology and achieving the Zero Hunger goal outlined in the United Nations Sustainable Development Goal 2. Among these, agricultural modernization has facilitated the adoption of biotechnology and increasingly efficient farming practices, enhancing crop yields while minimizing environmental impact in a sustainable manner.^46,47^

In this context, domestic sewage has been investigated as a sustainable source of P in WWTPs in many countries.^48–50^ It is estimated that around 17 to 31% of the P extracted from phosphate rock will be replaced by the recovered element by 2030 in the European Union. Forecasts suggest that by 2035, the demand for P will be greater than its availability, highlighting the need for its sustainable use and more efficient recovery methods.^51^ Phosphorus is a key component of various biomolecules including nucleic acids, nucleotides, phospholipids, and sugar phosphates. These molecules play essential roles in genetic information processing, energy transfer, and cellular structure,^52,53^ underscoring the significance of P in biological systems. This study focused on TechPhos, a novel selective adsorbent developed in Brazil for municipal wastewater treatment. Our results demonstrated its nontoxicity to lettuce, soy, and rice—three globally important crops. To better evaluate the seed response to TechPhos, germination-related indices were calculated. The GI is widely regarded as a reliable metric for assessing the phytotoxic potential of environmental samples.^61^ GI values equal to or greater than 80 generally indicate the absence or minimal presence of phytotoxic compounds. Values between 50 and 80 suggest a moderate level of phytotoxicity, whereas values below 50 are indicative of a substantial presence of phytotoxic agents.^54–56^ Based on these criteria, it can be concluded that 0.4 g L^–1^ TechPhos was not phytotoxic to *O. sativa*, *L. sativa*, or *G. max* seeds, as all of them achieved GI values above 80. This concentration was sufficient to confirm its safety while also promoting germination and growth in all three species. The chemical composition of certain nanofertilizers plays a crucial role in seed and plant development by supplying essential nutrients and supporting key physiological processes such as cell division, germination, and growth, including root and hypocotyl elongation. In particular, the beneficial effects of TechPhos may be linked to the absorption of micronutrients by soy, rice, and lettuce cells. The chemical compositions of Mnt and TechPhos revealed that both contain vital components involved in cell division and early developmental processes, such as K_2_O, MgO, CaO, Cl^–^, and Fe_2_O_3_, acting as a source of K, Mg, Ca, Cl, Fe, and Si.

K_2_O provides potassium, a vital macronutrient for plants, supporting cell expansion, turgor regulation, and stress resistance, as well as facilitating nutrient and sugar transport throughout the plant.^57–59^ MgO supplies magnesium, which is essential for chlorophyll formation, enzyme activation, and energy metabolism; magnesium also stabilizes ribosomes and cell membranes, ensuring efficient protein synthesis and cellular integrity.^57,60,61^ CaO, as a source of calcium, is crucial for cell wall structure by stabilizing pectins, supports cell division and elongation, and acts as a secondary messenger in signaling pathways related to stress and hormone responses; calcium also helps maintain membrane permeability and can mitigate toxic effects of certain soil elements.^62,63^ Chloride (Cl^–^) is involved in osmoregulation, stomatal function, and seed germination by aiding water uptake and cell expansion.^64^ Fe_2_O_3_ provides iron, which is indispensable for respiration, chlorophyll synthesis, and the activity of enzymes involved in energy production and seed development.^65^ Silicon (SiO_2_), while not essential, is recognized as a beneficial element that enhances plant resilience to stress, improves nutrient uptake, and supports structural integrity, with documented positive effects on crop performance.^66,67^ Together, these nutrients play interconnected roles in plant growth, development, and adaptation to environmental challenges, highlighting the importance of balanced mineral nutrition for optimal plant health and productivity.^58–61,68^

Many nanomaterials were recently studied toward understanding their role in improving agriculture production. Among these, Mnt and other clays have emerged as particularly promising due to their high surface area and cation exchange capacity, making them effective carriers of various substances. Their interlayer galleries provide space for active molecules, while inorganic sheets protect against rapid environmentally degradation. This unique combination has positioned nanoclays, especially Mnt, at the forefront of innovative applications for controlled delivery of fertilizers, growth plant regulators, and pesticides.^69^

In this work, we used bentonite rocks composed predominantly of the clay mineral montmorillonite, which belongs to the smectite group and is known for its ability to swell when in contact with water. Based on the results presented, the physicochemical properties of the iron-modified clay TechPhos indicate a strong potential for adsorption and ion exchange applications, particularly for nutrient and contaminant removal from aqueous media. The bentonite structure consists of stacked octahedral and tetrahedral silica sheets held together by van der Waals forces. The bentonite used in this study contains calcium as the predominant exchangeable cations, which are electrostatically bound and serve to balance the negative charges within the solid.^70,71^ The modification of natural montmorillonite clay with iron demonstrates a successful cation exchange process. The structural contraction observed indicates the effective ion exchange of Ca­(II) by Fe­(III) within the interlayer spaces, a hallmark of smectite-type clays. Conversely, the natural clay MnT exhibited no capacity to retain phosphate anions as it lacks adsorption sites with affinity for these species. Effective adsorption sites were generated following only the modification procedures, which resulted in the production of TechPhos.

Several key properties reveal an enhancement in TechPhos’s adsorptive performance: an increase in the surface area provides more active sites, and increased pore volumes indicate the generation of additional micro- and mesoporous structures, which are highly favorable for the diffusion and capture of adsorbate species, particularly in aqueous systems. These structural changes also result from removing accessory minerals.^72^ Type IV isotherms with H_3_ hysteresis loops are consistent with lamellar, slit-like pores, typical of clays, advantageous for adsorbing large or multivalent anions such as phosphate.

The physicochemical characterization confirms that TechPhos exhibits enhanced ion exchange capacity due to Fe­(III) doping and improved adsorption potential owing to its increased surface area and porosity. The XRF data confirm the effectiveness of the ion exchange process, particularly the successful replacement of Ca­(II) with Fe­(III). This chemical shift modifies the surface chemistry and potentially introduces active sites with a higher binding affinity for anions like phosphate. These changes significantly enhance the ion exchange capacity of TechPhos, especially toward anionic species due to the creation of more positively charged sites. Even prior to the ion exchange process, bentonite exhibits a high iron content due to its geological composition. Iron may be present either as iron oxide (an accessory mineral) or incorporated within the structural interstices of the bentonite, acting as an exchangeable or structural cation. The observed increase in iron content and the corresponding decrease in calcium content following ion exchange confirm the effectiveness of the process. These characteristics suggest that TechPhos is a promising material for environmental applications, especially for the removal of nutrients such as phosphate from wastewater via combined adsorption–ion exchange mechanisms.

The potential of using adsorbents as biofertilizers was highlighted by many authors.^73–78^ Carbon nanomaterials, silicates, and clays have been successfully tested in the wastewater remediation field.^79–81^ This approach enhances nutrient uptake, reduce fertilizer consumption, and contribute to agricultural production.^82,83^

Many studies suggest that montmorillonite enriched with iron could improve lettuce, soy, and rice growth by enhancing nutrient availability, promoting root development, and reducing the level of toxic compound accumulation without causing phytotoxicity.

Our results indicate that TechPhos did not exhibit toxic effects on the seedlings. TechPhos is composed of montmorillonite enriched with iron and municipal wastewater sludge absorbed into its matrix. As there are no prior reports evaluating the effects of TechPhos itself, we considered the available literature on its individual components separately. Regarding municipal sludge alone, previous studies have demonstrated its potential to enhance plant growth due to its high content of essential nutrients, such as nitrogen, phosphorus, and potassium. These nutrients are often found in higher concentrations than in conventional irrigation water and can reduce the reliance on synthetic fertilizers by up to 50% without compromising crop yields, as reported by Day and Ludeke^84^ and by Al-Suhaibani et al.^85^ In terms of montmorillonite alone, numerous studies have evaluated its impact on soil quality and plant health. For instance, Wang et al.^86^ showed that the inclusion of montmorillonite clay in soil significantly and consistently reduced the bioavailability of glyphosate and aminomethylphosphonic acid to corn roots and leaves in a dose- and time-dependent manner, without any observable toxic effects. Similarly, Hearon et al.^87^ investigated the behavior of per- and polyfluoroalkyl substances (PFAS) and their uptake by plants. Soil treatments with 2% montmorillonite-based sorbents significantly reduced PFAS bioavailability (up to 74%), and cucumber plants grown in these soils exhibited significantly lower PFAS residues (*p* ≤ 0.05). Furthermore, safety assessments using the macrophyte *Lemna minor* demonstrated that clays at a 0.1% inclusion rate enhanced growth compared to untreated controls. Zhang et al.^88^ studied the effect of montmorillonite-enriched siltstone (MS) on the physical properties of sandy soils and its influence on plant growth. MS addition significantly improved soil physical quality indicators, such as available water capacity and temporal stability, while decreasing the hydraulic conductivity and bulk soil air capacity. These improvements were attributed to the high content of the fine montmorillonite particles. Notably, aboveground biomass and water use efficiency increased by 2- to 5-fold, confirming the beneficial role of MS in improving sandy soil conditions. More recently, Cyriac et al.^89^ evaluated zinc-exchanged montmorillonite (Zn-MMT) as a slow-release nanofertilizer for rice cultivation. The effective intercalation of zinc within the montmorillonite layers led to significantly improved plant height, leaf area index, dry matter accumulation, number of tillers per hill, panicle length, and overall grain and straw yields compared to conventional zinc sulfate (ZnSO_4_) treatment. Zn-MMT-treated plants also exhibited higher levels of total phenols, proteins, chlorophyll, and phytochemicals such as indole acetic acid, superoxide dismutase, and carbonic anhydrase. Moreover, rice grains harvested from Zn-MMT-treated crops contained significantly higher zinc concentrations, confirming the enhanced nutrient-use efficiency provided by this slow-release formulation.

By extracting nutrients—primarily nitrogen and phosphorus—from sewage treated by sanitary plants using TechPhos, the residue can be transformed into a reusable input for nutrient release and fertilizer production. The resulting effluent, with a lower nutrient content, can be incorporated into the environment, thus reducing the environmental footprint. In bench tests, it was possible to achieve phosphorus removals as high as 90.3%. This level of removal provides disposal of final effluent containing phosphorus concentrations lower than 0.4 mg L^–1^, a strategic condition for maintaining quality of water resources, in relation to the serious problem of eutrophication, and reduces dependence on conventional fertilizers in agriculture.

Taken together, our results and the supporting literature suggest that TechPhos holds promise as a novel soil amendment with both environmental and economic advantages. Its application has the potential to contribute to sustainable agricultural practices by reducing the risk of eutrophication and supporting circular bioeconomy strategies, thereby offering broader benefits for the agri-food sector

In summary, our results indicate that TechPhos is nontoxic to the three species tested, enhances germination and root growth in *L. sativa* and *G. max* seeds, and serves as a fertilizer offering dual benefits: reducing nutrient pollution and strengthening its potential for agricultural applications. The proposal suggests the combination of TechPhos with sewage treatment plant sludge to create an innovative slow-release nutrient delivery system. This approach aims to provide an alternative for effluent treatment while simultaneously enhancing soil fertilization.

## 5 Conclusions

Our findings suggest that TechPhos, at the tested concentration, is nontoxic to soy, rice, and lettuce. Notably, it enhances germination and root growth in these species with the most significant effects observed in *G. max*, followed by *L. sativa* and *O. sativa*.

The study contributes to understanding TechPhos and wastewater sludge, as potential derivatives in agriculture, and highlights the need for systematic investigations to optimize their application to improve germination processes in agricultural activities, thereby presenting a potential sustainable solution in agricultural contexts.
